# Stop codon reassignment to tryptophan in members of the bacterial phylum Actinomycetota

**DOI:** 10.1099/mgen.0.001767

**Published:** 2026-08-10

**Authors:** Donovan H. Parks, Pierre-Alain Chaumeil, Maria Chuvochina, Philip Hugenholtz

**Affiliations:** 1 Aalborg University, Department of Chemistry and Bioscience, Center for Microbial Communities, Aalborg, Denmark; 2 The University of Queensland, School of Chemistry and Molecular Biosciences, Australian Centre for Ecogenomics, Brisbane, Queensland, Australia

**Keywords:** stop reassignment, genetic code, *Actinomycetota*, *Eggerthellaceae*, metagenome-assembled genomes

## Abstract

Reassignment of stop codons is a significant evolutionary event with recoding of UGA to tryptophan being previously identified in only three bacterial phyla, the *Bacillota*, *Pseudomonadota* and *Verrucomicrobiota*. Here, we present genomic evidence of this reassignment in a fourth bacterial phylum, the *Actinomycetota*, specifically in the family *Eggerthellaceae*. We identify the UGA stop-to-tryptophan reassignment in 34 metagenome-assembled genomes recovered from the stool samples of diverse mammalian hosts, including equids and primates. Canonical markers for this reassignment are consistently observed including conserved UGA codons aligning to tryptophan, loss of release factor 2 (*prfB*) and presence of a tRNA^Trp^(UCA) gene. We infer that this reassignment occurred at least twice as the lineages containing reassigned genomes are paraphyletic, forming two distinct groups separated by a third lineage with strains that use UGA as a stop codon. These lineages represent three new *Eggerthellaceae* genera for which we propose the type species *Equivita altericodex*, *Gorillivita intestinalis* and *Tapirivita inops* reflecting isolation source and genomic properties. Organisms representing these genera have reduced genomes and complete or partial loss of biosynthetic pathways, suggesting increasing host dependency and a transition to obligate symbiosis. This likely facilitated stop codon reassignment in *Equivita* and *Gorillivita* and suggests *Tapirivita* is primed for reassignment. This work expands the known phylogenetic diversity of UGA stop-to-tryptophan reassignment in the bacterial domain and establishes the *Eggerthellaceae* as a new focal point for understanding the evolutionary drivers of genetic code plasticity.

**Impact Statement**

While stop codon reassignment is a significant evolutionary event observed across domains, its phylogenetic distribution across the bacterial domain remains incompletely mapped. Here, we report the first genomic evidence of UGA stop-to-tryptophan reassignment within the phylum *Actinomycetota*, specifically in mammalian gut symbionts of the family *Eggerthellaceae*. We demonstrate that this recoding arose independently at least twice within this family alongside genomic reduction, consistent with the link between genetic code plasticity and transition to an obligate host-associated lifestyle. Our findings establish *Eggerthellaceae* as a compelling model system for understanding the evolutionary drivers of genetic code reassignment.

## Data Availability

The 35 MGR MAGs were submitted to the NCBI under BioProject PRJNA1366607. These MAGs were recovered from SRA samples (Table S1) and come from 13 BioProjects: PRJEB47977 (7 MAGs; unpublished), PRJEB73511 (1 MAG) [1], PRJNA1089803 (1 MAG) [1], PRJNA382701 (1 MAG) [2], PRJNA545604 (1 MAG; unpublished), PRJNA554776 (8 MAGs) [3], PRJNA635116 (3 MAGs) [4], PRJNA784453 (4 MAGs) [5], PRJNA814825 (2 MAGs) [6], PRJNA860652 (1 MAG) [7], PRJNA871798 (1 MAG; unpublished), PRJNA887424 (1 MAG) [8], and PRJNA983076 (4 MAGs) [9]. All unpublished data was submitted to the NCBI at least 3 years prior to this manuscript. This manuscript adheres to the data reuse guidelines of Hug et al. [10].

## Introduction

All organisms rely on a genetic code that translates nucleotide triplets (codons) on mRNA into specific amino acids or stop signals during protein synthesis [11]. Although this code is highly conserved, multiple independent reassignments have evolved in both cellular and organellar lineages [12]. Notably, several of these variations involve reassignment of stop codons to amino acids, a change identified in eukaryotic nuclear genomes [13, 14], mitochondria [15] and bacteria [16].

In bacteria, more than 99% of known species use the standard bacterial genetic code (code 11 https://www.insdc.org/submitting-standards/genetic-code-tables, hereafter GC11). Two variants of this code are known, both involving reassignment of the UGA stop codon to either tryptophan (code 4) or glycine (code 25). Reassignment of UGA to tryptophan has been identified in obligate intracellular endosymbionts belonging to three bacterial phyla: the *Bacillota* order *Mycoplasmatales* mainly comprising endosymbionts of vertebrates, arthropods and plants [17, 18]; *Pseudomonadota* insect endosymbionts *Candidatus* (*Ca*.) Hodgkinia cicadicola [19], *Ca*. Zinderia insecticola [20], *Ca*. Nasuia deltocephalinicola [21] and *Ca*. Stammera capleta [22] and the dinoflagellate parasite *Fastidiosibacteraceae* species XS4 [23]; and two *Verrucomicrobiota* ciliate endosymbionts *Ca*. Organicella extenuata [24] and *Ca*. Pinguicoccus supinus [25]. Reassignment of UGA to glycine has been observed in all known species of *Ca*. Absconditabacteria [26] and *Ca*. Gracilibacteria [27], with both groups inferred to be symbionts based on the lack of biosynthetic pathways for key metabolites [28, 29].

Bacterial genomes with UGA stop codon reassignment have several genomic features in common. This includes AT or GC compositional bias [19, 30] and reduced genome size (<2 Mb) [12], with the insect and dinoflagellate endosymbionts having highly reduced genomes (<500 kb) [20, 23]. However, the key molecular signature of a reassigned UGA stop codon is the loss of peptide chain release factor RF2 and the mutation of the tryptophan or glycine tRNA gene to recognize the UGA codon [19, 26]. In the standard bacterial code, the three stop codons (UAA, UAG and UGA) are recognized by peptide chain release factors RF1 and RF2, with RF1 recognizing UAA and UAG and RF2 recognizing UAA and UGA [31]. Because both RF1 and RF2 recognize UAA, organisms that have reassigned UGA generally no longer require RF2 and thus typically lack the *prfB* gene that encodes RF2 [32]. Translation of UGA as an amino acid canonically requires a tRNA with the UCA anticodon. Accordingly, most organisms using genetic code 4 (GC4) or 25 have a tRNA^Trp^(UCA) or tRNA^Gly^(UCA) gene, respectively. However, not all species with a UGA-to-tryptophan reassignment have a tRNA^Trp^(UCA) gene. It has been experimentally demonstrated using the parasitic protist *Blastocrithidia nonstop* that a mutation of the anticodon stem of tRNA^Trp^(CCA) from 5 to 4 bp enables efficient translation of UGA [33]. This 4 bp anticodon stem tRNA^Trp^(CCA) gene was subsequently identified in the *Fastidiosibacteraceae* bacterium XS4, which uses GC4 but lacks tRNA^Trp^(UCA) [23]. The functional versatility of UGA is further underscored by its role in encoding selenocysteine – the 21st amino acid – in a subset of organisms across all domains of life [34].

The phylogenetic distribution of UGA stop reassignments across the bacterial domain remains incompletely mapped. We identified three genomes in the *Actinomycetota* family *Eggerthellaceae* with a UGA stop codon reassignment during the initial development of a machine learning tool for predicting prokaryotic translation tables. Here, we provide genomic and phylogenetic evidence that the UGA codon has been reassigned to tryptophan in two *Eggerthellaceae* genera, a group of organisms commonly found in the gastrointestinal tract of humans and animals [35]. By analysing a broad range of *Eggerthellaceae* genomes, we show that UGA reassignment has occurred at least twice in this family accompanied by loss of multiple biosynthetic pathways, which we hypothesize indicates transition to an obligate host-associated lifestyle. This finding broadens the known taxonomic range of UGA reassignment and establishes a new bacterial family for exploration of the mechanisms and evolutionary consequences of genetic code reassignments.

## Results

### Recovery of *Eggerthellaceae* MAGs

The reassignment of the UGA stop codon to tryptophan in two as-yet-uncultured *Eggerthellaceae* genera, JAUNQF01 and CAVGFB01, was identified as a result of applying the genetic code prediction tools Codetta [16] and gTranslate [36] to genomes in GTDB release R10-RS226 [37]. To recover additional *Eggerthellaceae* genomes with this reassignment, Sandpiper [38] was used to identify metagenomic samples in the SRA [39] where organisms belonging to JAUNQF01 (407 samples) or CAVGFB01 (31 samples) were estimated to be at ≥1× coverage and ≥0.1% abundance (Table S2). Assembly and binning of these samples resulted in the recovery of 35 MAGs estimated to be ≥70% complete and ≤10% contaminated, with all but three (MGR1, MGR3 and MGR6) predicted to use GC4 (Tables 1 and S1).

**Table 1.** Genomic properties of *Eggerthellaceae* MAGs belonging to the genera JAUNQF01 and CAVGFB01, including 35 MAGs recovered from mammalian stool samples as part of this study

MAG ID	Genus	Species cluster*	Genetic code	Genome size (bp)	GC (%)	No. of contigs	16S rRNA†	No. of tRNA	Comp.‡ (%)	Cont.‡ (%)	Reference
GCA_030536315.1	JAUNQF01	8	4	1,089,702	39.2	42		20	79.97	0.09	[9]
GCA_030537355.1	JAUNQF01	2	11	1,514,811	35.1	21	Partial	20	93.05	0.22	[9]
GCA_963628375.1	CAVGFB01	1	4	1,044,520	32.0	158	Partial	17	88.2	0.81	[99]
MGR1	JAUNQF01	10	11	1,445,008	35.1	15		20	97.94	0.16	Present study
MGR2	CAVGFB01	1	4	999,203	31.8	51		18	92.27	0.0	Present study
MGR3	JAUNQF01	2	11	1,524,775	35.1	21	Partial	20	93.05	0.22	Present study
MGR4	JAUNQF01	4	4	1,400,689	37.8	119	Complete	18	92.34	0.09	Present study
MGR5	JAUNQF01	4	4	1,404,037	37.8	120	Complete	18	92.45	0.12	Present study
MGR6	JAUNQF01	2	11	1,523,567	35.1	23	Complete	20	93.05	0.24	Present study
MGR7	JAUNQF01	11	4	1,456,772	36.1	34	Complete	20	90.46	0.06	Present study
MGR8	CAVGFB01	1	4	1,220,050	32.1	91		15	91.5	0.46	Present study
MGR9	JAUNQF01	12	4	1,378,856	36.1	33	Partial	20	89.43	0.05	Present study
MGR10	JAUNQF01	8	4	1,246,223	39.0	42		18	88.82	0.04	Present study
MGR11	JAUNQF01	7	4	1,605,346	37.5	20		20	91.61	1.21	Present study
MGR12	JAUNQF01	7	4	1,391,096	38.0	38		19	85.56	0.21	Present study
MGR13	JAUNQF01	5	4	1,007,078	39.9	144	Partial	19	84.99	0.21	Present study
MGR14	CAVGFB01	1	4	1,429,972	31.3	81		19	95.92	2.79	Present study
MGR15	JAUNQF01	13	4	1,312,880	36.4	42	Partial	17	84.6	0.68	Present study
MGR16	JAUNQF01	14	4	1,599,280	37.1	32		18	80.74	0.14	Present study
MGR17	JAUNQF01	6	4	985,589	39.1	59		19	86.23	1.49	Present study
MGR18	JAUNQF01	9	4	1,042,509	39.1	153		18	80.71	0.45	Present study
MGR19	JAUNQF01	15	4	1,123,380	33.3	57		15	79.38	0.34	Present study
MGR20	JAUNQF01	3	4	1,347,907	37.4	182		18	81.49	1.05	Present study
MGR21	CAVGFB01	1	4	814,140	31.7	70		19	78.05	0.52	Present study
MGR22	JAUNQF01	16	4	1,217,471	38.8	80		19	77.03	0.42	Present study
MGR23	JAUNQF01	4	4	1,576,414	37.6	76		18	93.84	4.02	Present study
MGR24	JAUNQF01	3	4	1,433,483	37.0	138		15	75.19	0.32	Present study
MGR25	JAUNQF01	6	4	939,565	38.9	151		18	73.82	0.15	Present study
MGR26	CAVGFB01	17	4	1,491,620	39.4	281		18	74.17	0.46	Present study
MGR27	JAUNQF01	8	4	1,333,865	39.1	66		20	88.84	3.43	Present study
MGR28	JAUNQF01	18	4	1,849,454	32.1	81	Partial	18	89.26	3.63	Present study
MGR29	JAUNQF01	19	4	1,040,552	39.6	70		17	74.68	1.06	Present study
MGR30	JAUNQF01	9	4	1,085,064	39.0	93		19	77.23	2.55	Present study
MGR31	JAUNQF01	20	4	1,921,269	34.3	84		16	89.61	5.67	Present study
MGR32	JAUNQF01	21	4	1,170,523	38.9	126		18	72.52	2.52	Present study
MGR33	JAUNQF01	5	4	1,142,635	39.0	110		15	81.83	4.48	Present study
MGR34	JAUNQF01	22	4	1,202,115	37.5	97		17	76.42	5.04	Present study
MGR35	Novel	23	4	1,065,035	37.1	199		16	74.03	4.57	Present study

*Genomes belonging to the same species were established using a ≥95% average nucleotide identity threshold.

†Only considered complete if Prokka identified a start and stop codon for a 16S rRNA gene.

‡Comp., completeness; Cont., contamination; estimated using CheckM v2 [67].

**Fig. 1.**
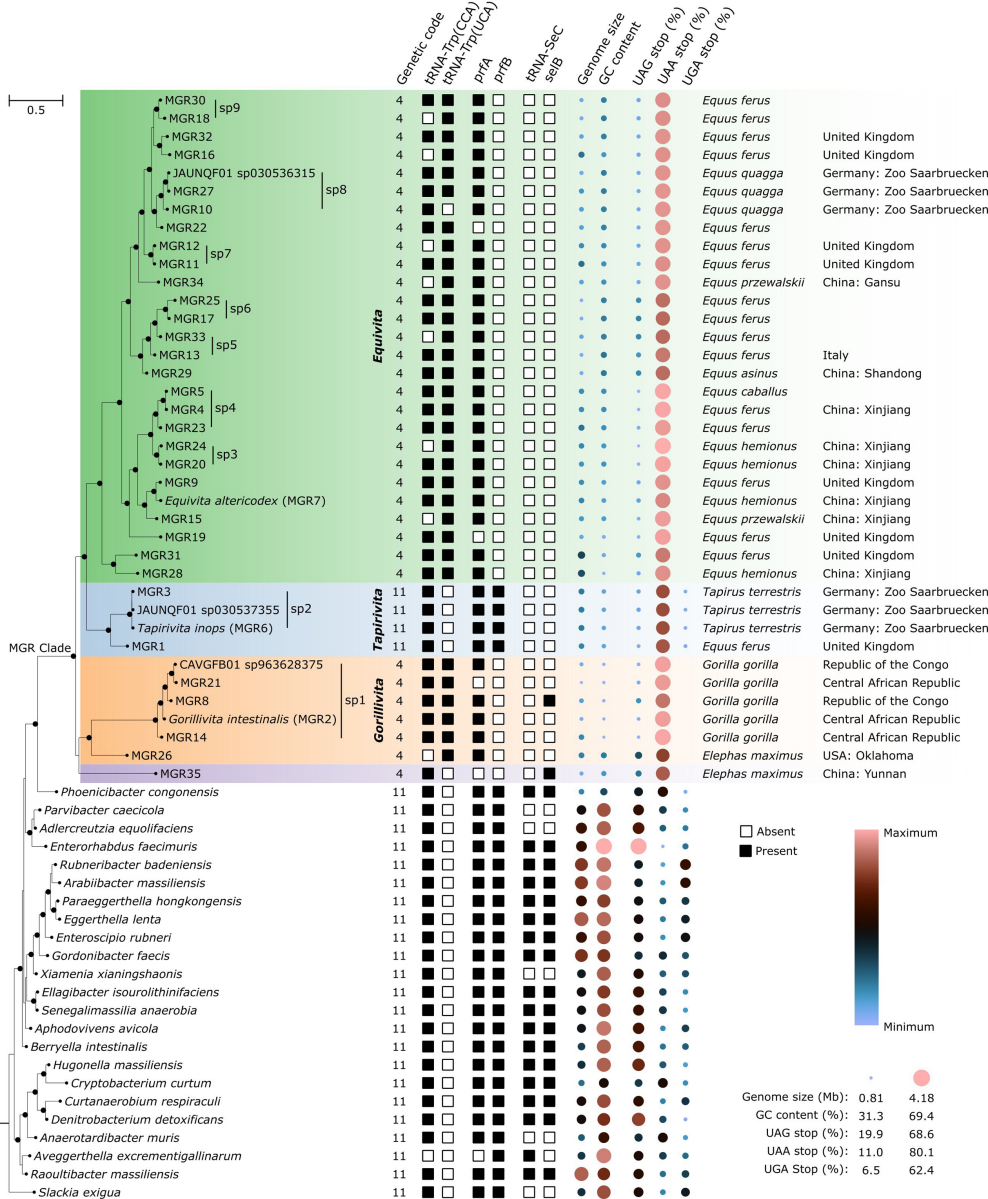
Maximum-likelihood phylogenetic tree inferred with IQ-TREE and the LG+F+C40 evolutionary model from an alignment of 120 bacterial marker genes, resulting in 30,832 informative sites after filtering of poorly aligned sites with TrimAl and compositionally biased sites with WitChi. *Equivita* MAGs (green shading), *Tapirivita* MAGs (blue shading), *Gorillivita* MAGs (orange shading) and a single reassigned MAG belonging to a new genus (purple shading) are shown along with isolate genomes from *Eggerthellaceae* genera. Genomes belonging to the same species are denoted by black vertical lines. The tree was rooted on the UBA8131 species WRGR01 sp009787085 (GCA_009787085.1) as this family is consistently found to be sister to *Eggerthellaceae*. Black dots on internal branches indicate strong support with UFBoot ≥95% and SH-aLRT ≥80%. Genomic properties that indicate a UGA stop to tryptophan reassignment are shown for each genome. Minimum and maximum values for genome size, %GC content and stop codon usage were calculated over all MAGs in the figure and isolate genomes from 61 *Eggerthellaceae* species in GTDB R10-RS226. *prfA*, peptide chain release factor RF1; *prfB*, peptide chain release factor RF2; *selB*, selenocysteine-specific elongation factor.

The 35 recovered *Eggerthellaceae* MAGs were classified using GTDB-Tk [40] with 29 and 5 assigned to JAUNQF01 and CAVGFB01, respectively, and 1 comprising a new genus in this family (Fig. 1, Table 1). These 35 MAGs, together with a single CAVGFB01 and 2 JAUNQF01 MAGs in GTDB R10-RS226, were grouped into 23 species clusters using a 95% average nucleotide identity criterion. Nine of the species clusters contained multiple MAGs, with only cluster sp5 (comprised of MGR13 and MGR33) not being resolved as monophyletic (Fig. 1, Table 1). The 38 MAGs were recovered from mammalian stool samples, so we refer to these as the mammalian gut reassignment (MGR) clade and to the paraphyletic subsets of MAGs predicted to use GC4 as MGR-GC4.

### MGR genomes represent new genera and species in *Eggerthellaceae*

Here, we propose type genomes and names for MGR taxa following the SeqCode [41], a sequence-based nomenclatural code. We propose that the JAUNQF01 genus within the family *Eggerthellaceae* be divided into two genera, one comprising MAGs using genetic code 11 and the other MAGs using GC4 (Fig. 1). The proposal for two genera is supported by phylogenetic trees (*see below*), relative evolutionary divergence [42], and the percent identity of 16S rRNA gene sequences [43] in these two clades (see Note S1). We designate MGR6 as the type genome of the type species for the JAUNQF01 GC11-clade and propose the name *Tapirivita inops* gen. nov., sp. nov. to reflect the host of this MAG and its reduced genome size. For the GC4-clade of JAUNQF01, we designate MGR7 as the type genome and propose the name *Equivita altericodex* gen. nov., sp. nov. to reflect the host of this MAG and reassignment of the UGA stop codon. Finally, we designate MGR2 as the type genome of the type species for the CAVGFB01 genus and propose the name *Gorillivita intestinalis* gen. nov., sp. nov. to reflect the host of this MAG and its association with the gastrointestinal tract of mammals. Protologues for new taxa are provided in Table S3. The MAGs selected as type genomes meet most criteria required to be considered high quality [44], as they have an estimated completeness >90% with minimal estimated contamination, are comprised of ≤51 contigs and have ≥18 tRNA genes (Table S1). However, MAGs often lack the rRNA operon, and this is reflected in the selected MAGs with only MGR6 containing the 5S, 16S and 23S rRNA genes, MGR7 having only a 16S rRNA gene and MGR2 missing all three of these rRNA genes.

### Phylogenetic relationship of *Eggerthellaceae* genomes

A maximum-likelihood tree was inferred to examine phylogenetic relationships among *Eggerthellaceae* genomes and the 38 MGR MAGs (Fig. 1, Table S1). Notably, the *Equivita* and *Gorillivita* MAGs form two distinct lineages that use GC4, indicating either two independent reassignment events or a single reassignment event followed by a reversion to GC11 in the *Tapirivita* lineage. To confirm these evolutionary relationships, 24 phylogenetic trees were inferred using varying marker sets, alignments, inference methods and evolutionary models (Table S4). All trees indicate that the MGR clade comprising *Equivita*, *Gorillivita*, *Tapirivita* and MGR35 is monophyletic (Figs 1 and S1). Notably, the GC4 genera *Equivita* and *Gorillivita* were consistently paraphyletic and separated by the GC11 genus *Tapirivita*. GTDB-Tk classified MGR35 as a new *Eggerthellaceae* genus consistent with its unstable phylogenetic placement: 14 of 24 trees placed it sister to *Gorillivita*, 9 placed it as the most basal lineage in the MGR clade and 1 placed it between *Gorillivita* and *Tapirivita* (Fig. S1). Most trees (20 of 24) place the MGR clade as a non-basal lineage within the *Eggerthellaceae* except for four trees inferred with a more heuristic inference method (FastTree) and simple evolutionary models (WAG or LG+G; Fig. S1).

### Genomic properties supporting codon reassignment

**Fig. 2.**
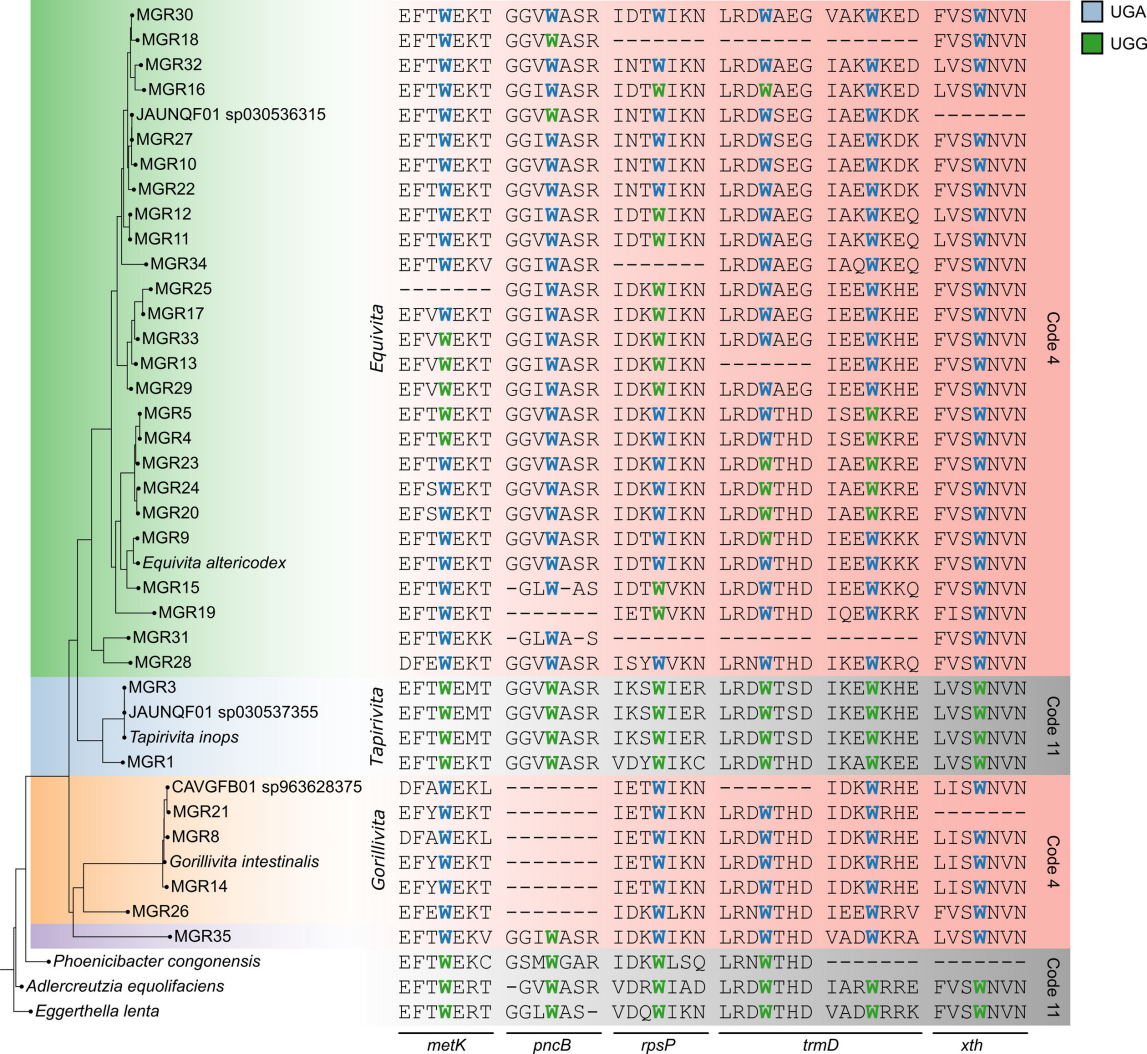
Aligned proteins indicating the UGA codon codes for tryptophan in the 27 *Equivita* MAGs, 6 *Gorillivita* MAGs and the MGR35 MAG. Conserved tryptophan (W) residues are encoded by UGA (blue) or UGG (green). Partial sequences are for proteins encoded by the genes *metK* (methionine adenosyltransferase), *pncB* (nicotinate phosphoribosyltransferase), *rpsP* (ribosomal protein S16), *trmD* (guanine(37)-N(1))-methyltransferase) and *xth* (exodeoxyribonuclease III).

UGA-to-tryptophan reassignment was predicted by two complementary tools: gTranslate, a machine learning method for inferring the genetic code used by prokaryotic genomes, and Codetta, a homology-based approach that predicts reassignments by examining alignments of conserved proteins. Across the 34 MGR-GC4 MAGs (i.e. *Equivita* MAGs, *Gorillivita* MAGs and MGR35), Codetta identified 249 to 823 (mean=488) instances where UGA aligned to a conserved tryptophan residue (Table S1). No additional deviations from the standard bacterial genetic code were identified by Codetta. We identified five proteins that illustrate the alignment of UGA codons to conserved tryptophan residues across the 34 MGR-GC4 MAGs contrasted to the 4 GC11 *Tapirivita* MAGs and 3 outgroup *Eggerthellaceae* genomes (Fig. 2).

The MGR-GC4 MAGs exhibit genomic properties that are typical of organisms with stop codon reassignment (Table 2). This includes having a relatively small genome size (mean=1.28 Mb) and low %GC content (mean=36.8%) in comparison to other *Eggerthellaceae* species (mean genome size=2.23 Mb; mean GC=58.6%; Table S1). All MGR MAGs have a homologue of *prfA* (RF1; Fig. 1), except for four that we attribute to these MAGs being incomplete (mean estimated completeness of 85.0±7.4%; Table S1). By contrast, all MGR-GC4 MAGs lack a homologue of *prfB* (RF2; Fig. 1) and sequence similarity searches against *Eggerthellaceae* RF2 proteins indicated the absence of even pseudogenized *prfB* sequences in these MAGs (see Methods). Loss of *prfB* is common in genomes using GC4, as this gene is dispensable when UGA no longer serves as a stop codon (Table 2) [17, 45]. Additionally, the majority of MGR-GC4 MAGs contain tRNA^Trp^ genes with both CCA and UCA anticodons (Fig. 1), the latter enabling recognition of UGA as tryptophan [46]. The presence of a tRNA^Trp^(UCA) gene is common among bacterial species using GC4 (Table 2), though not ubiquitous as exemplified by the *Fastidiosibacteraceae* XS4 bacterium [23].

**Table 2.** Genomic properties of selected bacterial taxa using GC4 Comparisons highlight that the *Eggerthellaceae* genera *Equivita* and *Gorillivita* share genomic characteristics with *Mycoplasmatales* and other GC4 endosymbionts. Genomes were sourced from NCBI RefSeq release 226. Calculations for *Mycoplasmatales* were restricted to type strain assemblies, while all available genomes were included for the candidate taxa *Ca*. Hodgkinia cicadicola, *Ca*. Nasuia deltocephalinicola, *Ca*. Stammera capleta or *Ca*. Zinderia insecticola.

	*Equivita* and *Gorillivita* spp.	*Mycoplasmatales* spp.	*Ca*. Hodgkinia cicadicola	*Ca*. Nasuia deltocephalinicola	*Ca*. Stammera capleta	*Ca*. Zinderia insecticola	*Ca*. Pinguicoccus supinus
No. of genomes	34	220	13	24	49	1	1
No. of species	21	160	1	1	1	1	1
%GC content	31.3 to 39.9 mean=36.8	22.4 to 40.0 mean=27.1	38.7 to 58.4 mean=47.6	15.2 to 18.0 mean=16.9	13.9 to 20.4 mean=16.9	13.5	25.1
Genome size (Mb)	0.81 to 1.92 mean=1.28	1.18 to 2.23 mean=1.34	0.11 to 0.15 mean=0.14	0.11 to 0.14 mean=0.11	0.22 to 0.34 mean=0.29	0.21	0.16
tRNA^Trp^(CCA)	27 of 34	211 of 220	No	No	Yes	Yes	No
tRNA^Trp^(UCA)	32 of 34	219 of 220	Yes	Yes	No	No	Yes
*prfB* (RF2)	No	No	No	No	15 of 49	No	No
tRNA-SeC	No	No	No	No	No	No	No
*selB*	1 of 34	2 of 220	No	No	No	No	No
UAA stop (%)	65.2 to 80.1 mean=75.6	65.5 to 91.3 mean=80.8	30.5 to 68.8 mean=55.0	87.1 to 96.6 mean=91.8	88.2 to 97 mean=92.8	95.8	78.5
Host	Mammalian gut	Vertebrates, arthropods, plants	Cicadas (*Cicadoidea*)	Leafhoppers (*Hemiptera*)	Leaf beetles (*Cassidinae*)	Spittlebugs (*Cercopoidea*)	Ciliate (*Euplotes vanleeuwenhoeki*)
Phylum	*Actinomycetota*	*Bacillota*	*Pseudomonadota*	*Pseudomonadota*	*Pseudomonadota*	*Pseudomonadota*	*Verrucomicrobiota*
Reference	This Study	[17]	[19]	[21]	[22]	[20]	[25]

*prfB*, peptide chain release factor RF2; *selB*, selenocysteine-specific elongation factor.

MGR-GC4 MAGs preferentially use the UAA instead of UAG stop codon (mean frequency of 75.9% vs 24.1%; Fig. 1, Table S1), presumably reflecting their low %GC content [47]. Notably, the GC11 *Tapirivita* MAGs also favour the UAA stop codon (mean=67.5%; minimum=66.7%) and rarely use the UGA stop codon (mean=9.2%; maximum=9.6%). This contrasts with other *Eggerthellaceae* species, where usage is more even across the three stop codons (UAG=44.6%, UAA=34.9% and UGA=20.5%; Fig. S2). The highest usage of UAA is 61.6%, and only 15 of 212 (7.1%) genomes exhibit UGA usage at or below 9.6% (Table S1). This suggests that the *Tapirivita* MAGs have experienced similar selective pressures as the MGR-GC4 MAGs that resulted in reduced %GC content, preferential usage of the UAA stop codon and reassignment of UGA to tryptophan.

Collectively, these results support UGA encoding tryptophan in members of the MGR clade, with the notable exception of the two *Tapirivita* species where genomic evidence indicates they use UGA as a stop codon.

### Evidence for multiple reassignment events

**Fig. 3.**
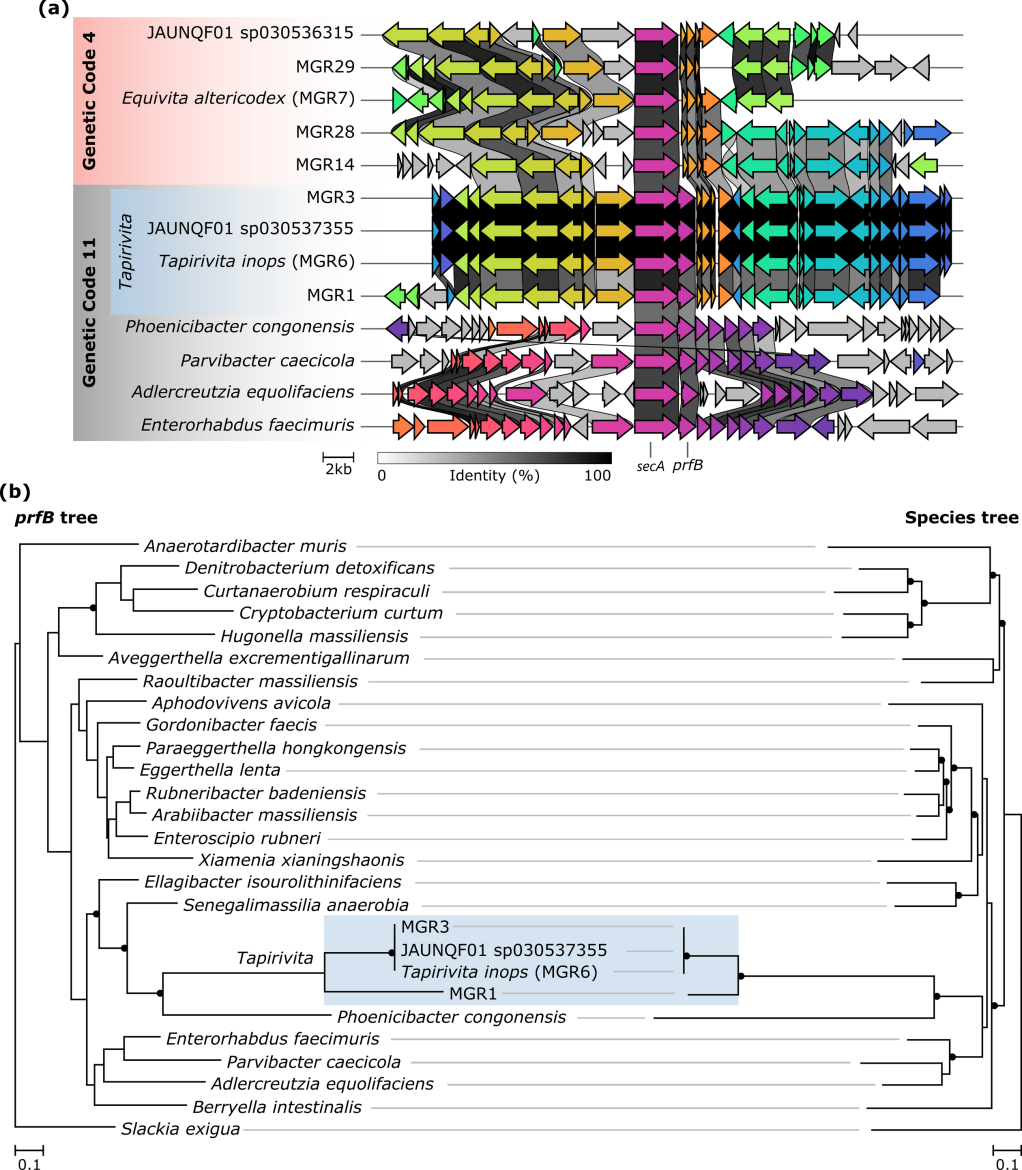
Gene neighbourhood of *prfB* (**a**) and the phylogeny of this gene (**b**) support two independent reassignment events. (**a**) Conservation of genes around *secA* including its close proximity to *prfB* (RF2) in *Eggerthellaceae* genomes using genetic code 11 suggest *prfB* was not lost and reacquired in *Tapirivita*. Genes in adjacent genomes with >35% identity are connected by a grey ribbon. (**b**) Tanglegram of the *prfB* gene tree and *Eggerthellaceae* species tree from Fig. 1. The *prfB* tree is a maximum-likelihood tree inferred with IQ-TREE and the Q.pfam+I+G4 evolutionary model. Black dots on internal branches indicate strong support with UFBoot ≥95% and SH-aLRT ≥80%. *prfB*, peptide chain release factor RF2; *secA*, protein translocase subunit.

Loss of *prfB* (RF2) has been proposed as the common force driving UGA stop reassignment events [19]. The loss of this gene is nearly universal in organisms that have reassigned the UGA stop codon as the presence of RF2 would result in early termination of proteins at UGA codons (Table 2) [32]. The phylogenetic relationship of MAGs in the MGR clade suggests two independent reassignment events or a single reassignment event followed by reversion to code 11 in the genus *Tapirivita* (Fig. 1). Examination of the gene neighbourhood around *prfB* (RF2) and the phylogeny of this gene support two independent reassignment events. The *secA* and *prfB* genes have previously been reported to constitute an operon in *Bacillus subtilis* [48], and we observed that these genes are adjacent (175 of 178 species) or occasionally separated by a single gene (3 of 178 species) in *Eggerthellaceae* species using genetic code 11 (Fig. 3a, Table S1). The conserved adjacency of *secA* and *prfB* in the *Tapirivita* MAGs suggests *prfB* was not lost in this lineage, as re-acquisition via horizontal gene transfer into this specific locus is unlikely. The *prfB* phylogeny further supports that this gene has not been lost by *Tapirivita* MAGs, as it is largely congruent with the species tree, noting that *Phoenicibacter congonensis* is the closest genetic code 11 species to *Tapirivita* MAGs in both trees (Figs 1 and 3b). A possible hybrid explanation may have involved an MGR ancestor with rare UGA usage, presence of the *prfB* and tRNA^Trp^(UCA) genes and ambiguous UGA translation. This could then have proceeded to complete reassignment in *Equivita* and *Gorillivita* by independent deletions of *prfB* and reversion to a stop codon in *Tapirivita* by loss of tRNA^Trp^ (UCA). In this scenario, we would expect to see conservation of the gene neighbourhood around tRNA^Trp^(UCA) in *Equivita* and *Gorillivita*; however, this was not the case (Fig. S3), further supporting two independent reassignment events. The implications of multiple reassignment events in this lineage are discussed in the next section.

### Inferred transition of MGR organisms to obligate host dependency

Stop codon reassignment is often associated with a transition to obligate host association as seen for *Mycoplasmatales* and *Pseudomonadota* endosymbionts [49]. Such transitions are marked by reductive evolution that typically results in reduced genome size and change in %GC content, as already noted for MGR genomes (Table 1) and loss of biosynthetic pathways and cell envelope structures [49]. Loss of biosynthetic capability is linked to availability and increasing reliance on host metabolites and deterioration of the cell envelope has been linked to osmotic homeostasis in a eukaryotic host cell and reduced immune triggering [50]. All MGR genomes (including the GC11 *Tapirivita* species) displayed complete or partial loss of biosynthetic pathways relative to other *Eggerthellaceae* genomes, including for amino acids (histidine, leucine, isoleucine, threonine, proline, lysine and arginine), vitamins (B6, B9) and core metabolism (fatty acid synthesis, tricarboxylic acid cycle; Table S5). By contrast, genes for synthesis and maintenance of the cell envelope remain mostly intact (Table S6), which has been observed in GC11 obligate insect endosymbionts including *Buchnera*, *Ca*. Blochmanniella and *Wigglesworthia* [51–53] and appears to be the case in at least one GC4 symbiont, *Ca*. Stammera capleta [22]. Together, these genomic features suggest that MGR clade species have undergone genome reduction and transitioned to a host-dependent lifestyle in the mammalian gut.

The presence of the GC11 genus *Tapirivita*, with hallmark features of obligate symbionts, in the midst of GC4 species in the MGR clade (Fig. 1) calls into question the timing of UGA reassignment. UGA to tryptophan reassignment is unlikely to be reversible [54, 55], suggesting that at least two independent reassignment events have occurred in the MGR lineage (see discussion above) in bacteria that had already undergone genome reduction. Therefore, stop codon reassignment is more likely to be a consequence of genome reduction and host adaptation rather than a cause. Low usage of UGA stop codons in the two *Tapirivita* species (~9%; Fig. 1) suggests that they may be primed for a reassignment event and thus may offer compelling targets for studying this phenomenon.

### Host association and biogeography of MGR genera

**Fig. 4.**
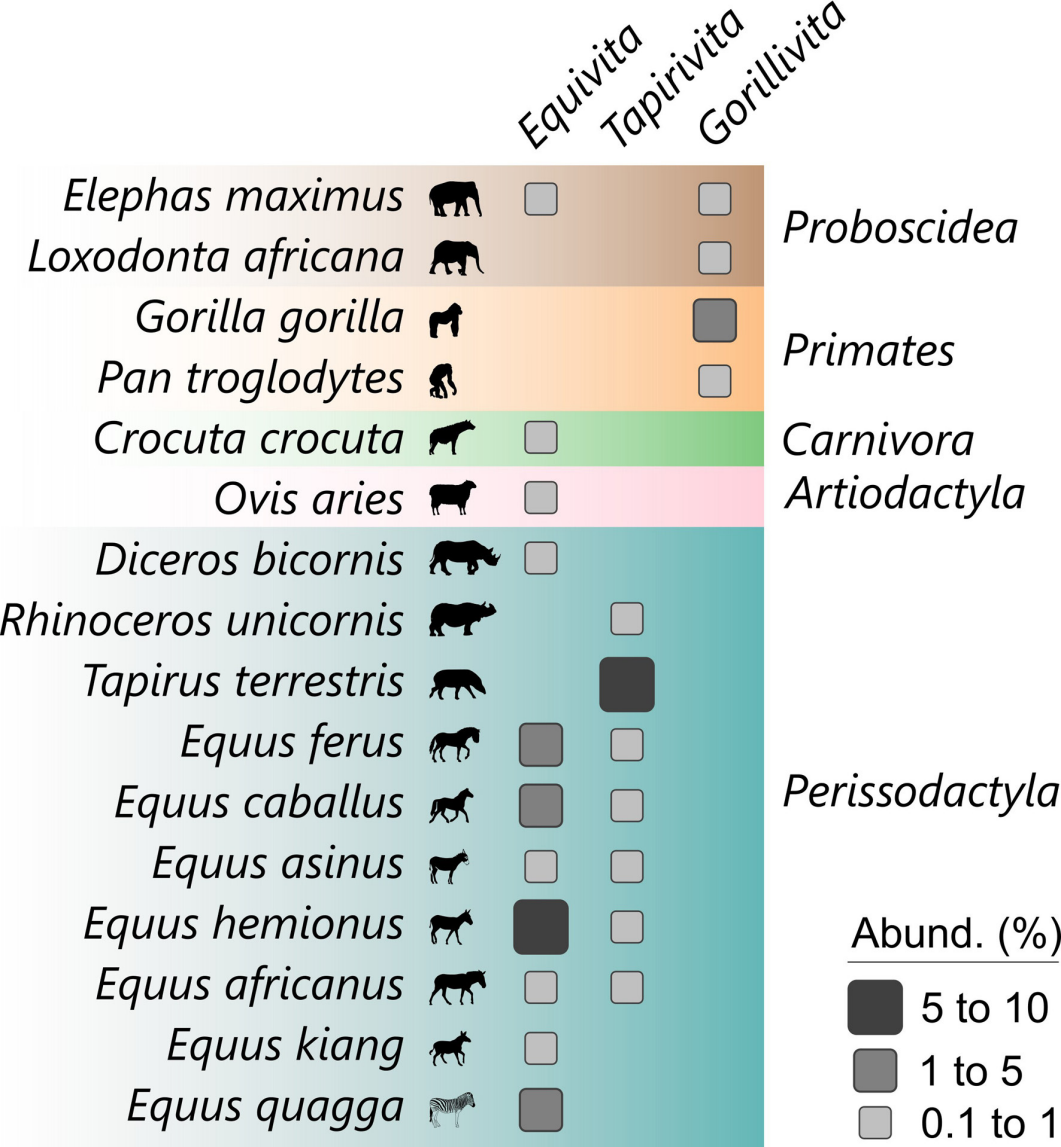
Hosts associated with the MGR genera *Equivita*, *Tapirivita* and *Gorillivita* identified by metagenomic profiling. The maximum relative abundance of MGR genera across profiled samples is indicated for each host species using grey boxes. Hosts are organized and coloured by taxonomic order.

Bacteria that use GC4 are typically symbiotic organisms with reduced genomes that reside in a range of arthropod, vertebrate, plant and protist hosts (Table 2). MGR organisms follow this pattern and were exclusively recovered from mammalian stool samples. *Equivita* MAGs were exclusively associated with hosts in the *Perissodactyla* genus *Equus* (e.g*.* horses and zebras; Fig. 1), but metagenomic profiling indicates a broader host range spanning the additional *Perissodactyla* species *Diceros bicornis* (black rhinoceros) along with *Ovis aries* (sheep), *Crocuta crocuta* (hyena) and *Elephas maximus* (elephant; Fig. 4, Table S2). By contrast, *Tapirivita* species may be restricted to hosts in the order *Perissodactyla* with MAGs recovered from *Equus* and *Tapirus terrestris* (tapir) stool samples and metagenomic profiling only increasing the host range to include the *Perissodactyla* species *Rhinoceros unicornis* (Indian rhinoceros). *Gorillivita* MAGs were recovered from *Gorilla gorilla* and *E. maximus* faecal samples, and metagenomic data expanded this host range to include *Pan troglodytes* (chimpanzee) and *Loxodonta africana* (African bush elephant). Notably, this suggests the *Gorillivita* species may not be found in *Perissodactyla* hosts.

*Equivita* and *Tapirivita* are likely widely distributed due to the geographic range of their mammalian hosts. Equine stool samples containing these MGR genera were collected in Europe, Asia and North America (Table S2). Samples with *Equivita* were also collected in South Africa, Kenya and Tanzania from *D. bicornis*, *C. crocuta* and *Equus quagga*, respectively. *Tapirivita* was also identified in a *R. unicornis* stool sample from Nepal and *T. terrestris* (tapir) samples collected from the Saarbrücken Zoo in Germany, suggesting that *Tapirivita* species may be present in South America, the tapir’s native habitat. The geographic range of *Gorillivita* includes Central Africa where it is associated with gorillas and/or chimpanzees (Table S2) and presumably India and Southeast Asia as these are the native ranges for *E. maximus* and *L. africana*, though these samples were obtained from the Oklahoma [8] and Beijing [56] zoos, respectively.

## Discussion

Bacteria using GC4 have previously only been identified in the phyla *Bacillota* [57], *Pseudomonadota* [20] and *Verrucomicrobiota* [24, 25]. The MGR-GC4 genera *Equivita* and *Gorillivita* belong to the phylum *Actinomycetota*, thereby expanding the number of phyla known to contain organisms using this reassignment. The average genome sizes of MGR-GC4 MAGs (mean=1.28 Mb) and GC4 *Bacillota* order *Mycoplasmatales* genomes (mean=1.34 Mb) are similar (Table 2) and, while small compared to most bacterial organisms (~5 Mb) [58], are much larger than the highly reduced GC4 *Pseudomonadota* genomes (<0.5 Mb) of insect or dinoflagellate endosymbionts [20, 23]. These organisms often have extremely low %GC content (Table 2) as exemplified by the insect endosymbionts *Ca*. Nasuia deltocephalinicola (mean=16.9%) [21], *Ca*. Stammera capleta (mean=16.9%) [22] and *Ca*. Zinderia insecticola (13.5%) [20] and to a lesser extent the dinoflagellate endosymbiont XS4 (28.2%) [23]. The majority of *Mycoplasmatales* species also exhibit low %GC content (mean=27.1%). However, *Ca*. Hodgkinia cicadicola with a %GC content as high as 58.4% [19] demonstrates that low %GC content is not a requirement of organisms that use GC4 and the MGR-GC4 MAGs with %GC ranging from 31 to 40% (mean=36.8%) support a wider %GC content range for GC4 organisms than previously appreciated.

While this study has primarily focused on evidence supporting a UGA reassignment and establishing the phylogenetic relationship between *Eggerthellaceae* species, the presence of both GC4 and GC11 species in the MGR clade makes it an interesting system for investigating the mechanisms leading to and the results of UGA being reassigned to tryptophan. However, any such investigations will benefit from the recovery of additional *Tapirivita* MAGs as this genus currently comprises only four MAGs from two species, with three of the four (MGR3, MGR6 and JAUNQF01 sp030537355) being recovered from biological replicates of, presumably, a single tapir at the Saarbrücken Zoo in Germany [9]. The majority of *Eggerthellaceae* species outside the MGR clade have the genes required to incorporate selenocysteine into proteins using a translational recoding of UGA, i.e*.* a selenocysteine-specific tRNA (tRNA^SeC^) and a specialized translation factor (*selB*) that bring Sec-tRNA^SeC^ to the ribosome at UGA sites with a downstream selenocysteine insertion sequence (SECIS) element (Fig. 1) [59, 60]. Consequently, the MGR clade being sister to these *Eggerthellaceae* species and lacking selenocysteine-associated genes suggests that translational recoding of UGA may have acted as a precursor to the MGR clade reassigning UGA to tryptophan. To our knowledge, the use of UGA for selenocysteine potentially priming UGA stop codon recoding has not been previously proposed, and the mechanisms that would support this change are unclear. One hypothesis is that selenocysteine incorporation competes with the RF2 release factor for the UGA codon [61, 62] resulting in a preference for alternative stop codons and a reduction in the use of RF2, ultimately resulting in the loss of UGA as a stop codon and the need for the RF2 gene.

The MGR-GC4 MAGs are broadly consistent with the ‘codon capture’ [30, 63], ‘ambiguous intermediate’ [15, 64, 65] and ‘unassigned codon’ [66] models for the reassignment of the UGA stop codon to tryptophan. The codon capture model proposes that this reassignment occurs after the UGA stop codon has been driven to extinction because of reduced %GC content that favours a mutational bias to the UAA stop codon. The UGA codon would then be under no pressure to be maintained as a stop codon and could be re-introduced as recognizing tryptophan. The MGR-GC4 MAGs are consistent with this model as they have lower %GC content (mean=36.8%) than other *Eggerthellaceae* species (mean GC=58.6%). While %GC content of the extant MGR-GC4 MAGs is arguably not sufficiently low to support the codon capture model, it is possible that the ancestor(s) of these extant species had substantially lower %GC content that would be compatible with this model, or another mechanism not reliant on low %GC caused the reassignment. The ambiguous intermediate model proposes that a single codon is temporarily read in two different ways, with a subsequent loss of the original meaning of the code. McCutcheon *et al*. [19] adapted this model to explain UGA reassignment in the high %GC *Ca*. H. cicadicola where a mutated tRNA^Trp^(CCA) would allow recognition of both UGG and UGA. This would be followed by loss of RF2 as the genome mutates to using UAA and UAG as stop codons and UGA as tryptophan. For *Ca*. H. cicadicola, subsequent mutation of the tRNA^Trp^ anticodon would result in these genomes exclusively having tRNA^Trp^(UCA). This model is largely consistent with the MGR-GC4 MAGs though these genomes have retained a tRNA^Trp^(CCA) gene (Fig. 1). This could be the result of an initial duplication of the tRNA^Trp^(CCA) gene, which is plausible as multiple copies of this gene were identified in 14 *Eggerthellaceae* species (Table S1). Alternatively, the unassigned codon model proposes that the loss of RF2 occurs before the gain of the tRNA^Trp^(UCA) gene. Given the lack of syntenic conservation of the tRNA^Trp^(UCA) gene between *Equivita* and *Gorillivita* (Fig. S3), the order of RF2 loss and gain of tRNA^Trp^(UCA) remains unclear, making both the ambiguous intermediate and unassigned codon models possible explanations for the observed reassignments.

## Conclusions

We present genomic evidence for UGA stop-to-tryptophan reassignment in 34 MAGs comprising 3 genera and 21 species in the family *Eggerthellaceae* and have proposed the genus names *Tapirivita* (formerly*,* JAUNQF01 in GTDB R10-RS226)*, Equivita* (formerly*,* JAUNQF01) and *Gorillivita* (formerly, CAVGFB01). These MAGs were found exclusively in mammalian stool samples, exhibit both host and geographic structuring and are the first members of the bacterial phylum *Actinomycetota* identified with this reassignment. They have features consistent with a transition to obligate host association but are notable for having moderate %GC content compared to the AT rich genomes typical of GC4 obligate symbionts, raising questions about the evolutionary mechanism that drove reassignment. We infer that UGA stop-to-tryptophan reassignment occurred at least twice in the *Eggerthellaceae* based on phylogenetic and gene synteny evidence that suggests this transition was a consequence of genome reduction and host adaptation rather than a driver, and that *Tapirivita* is primed for reassignment. Thus, the family *Eggerthellaceae* provides a new focus for furthering our understanding of the mechanisms leading to genetic code reassignments.

## Methods

### Recovery of additional *Eggerthellaceae* metagenome-assembled genomes

Metagenomic samples from the Sequence Read Archive (SRA) [39] likely to result in recovery of *Eggerthellaceae* genera CAVGFB01 or JAUNQF01 metagenome-assembled genomes (MAGs) were identified using Sandpiper 1.0.0 [38]. Specifically, we processed SRA runs where either genus was estimated to be at ≥1× coverage and ≥0.1% abundance with Aviary 0.12.0 (https://github.com/rhysnewell/aviary) using default parameters. This resulted in 438 samples (31 CAVGFB01; 407 JAUNQF01) being processed (Table S2, available in the online Supplementary Material). Recovered MAGs were classified using GTDB-Tk v2.4.1 [40], which resulted in the identification of 15 CAVGFB01 MAGs, 84 JAUNQF01 MAGs and a single MAG from a novel *Eggerthellaceae* genus predicted to use GC4. CheckM2 1.1.0 [67] was used to estimate the quality of these 100 MAGs and the 35 MAGs (5 CAVGFB01, 29 JAUNQF01 and the novel *Eggerthellaceae* MAG) with ≥70% completeness, ≤10% contaminated and a quality score ≥50 (defined as completeness−5×contamination) were used in subsequent analyses. These 35 MAGs came from 27 SRA runs, which have a minimum estimated CAVGFB01 or JAUNQF01 coverage of 6.8× and abundance of 0.17%.

Aviary used the following programs to recover MAGs: fastp 1.0.1 [68], metaSPAdes 4.0.0 [69], CoverM 0.7.0 [70], minimap2 2.18 [71], MetaBAT 2.15 [72], SemiBin2 2.2.0 [73], Vamb 5.0.4 [74], DAS Tool 1.1.2 [75], CheckM 1.2.4 [76], CheckM2 1.1.0 [67] and pplacer 1.1 [77]. The translation table of *Eggerthellaceae* genomes, including the 35 quality-filtered mammalian gut reassignment (MGR) MAGs, was predicted using gTranslate 0.0.2 [36] and Codetta 2.0 [16] using default settings.

### Assignment of MGR MAGs to species clusters

The 35 MGR MAGs along with the 2 JAUNQF01 and single CAVGFB01 MAGs in GTDB R10-RS226 were organized into operational species clusters using Galah 0.4.2 (github.com/wwood/galah). Galah uses the same representative selection criteria and clustering methodology as GTDB [78], except the presence of 16S rRNA genes is not considered.

### Inference of species trees

The phylogenetic relationship of *Eggerthellaceae* species was determined using genomes from GTDB R10-RS226 and the 35 MGR MAGs recovered in this study (Table S1). Specifically, trees were inferred using the 212 (of 297) GTDB species representatives within the *Eggerthellaceae* family with a CheckM2 completeness ≥80%, contamination ≤5% and ≤200 contigs along with the CAVGFB01 (GCA_963628375.1) and JAUNQF01 (GCA_030537355.1, GCA_030536315.1) genomes. The trees were rooted using the WRGR01 sp009787085 genome GCA_009787085.1, the highest-quality MAG in the family UBA8131, which is sister to *Eggerthellaceae* according to the GTDB R10-RS226 reference tree.

Trees were inferred using four marker sets, varying methods for producing and trimming the multiple sequence alignment (MSA), and different tree inference methods and models of evolution (Table S4). The four marker sets considered were the 120 bacterial (bac120) marker genes used by GTDB [79], a set of 16 ribosomal proteins (rp1) [80], a set of 23 ribosomal proteins (rp2) [27] and a set of 339 core *Eggerthellaceae* genes. The bac120 genes were identified using GTDB-Tk 2.4.1 [40]. The rp1 and rp2 genes were identified using EasyCGTree 4.2 [81] run with default values. The core *Eggerthellaceae* gene set was established using a modified version of Panaroo 1.5.2 [82] that allowed the genetic code for each genome to be individually specified. Panaroo was run in strict mode using a sequence identity threshold of 60%, protein family identity threshold of 50%, length difference threshold of 60% and core gene threshold of 70%. Genes were aligned using HMMER 3.4, MUSCLE 5.1 [83] run in ‘align’ mode, or MAFFT 7.505 [84] run using the L-INS-I alignment method and ‘maxiterate’ set to 1,000. Trees were inferred for each MSA without filtering, after filtering with TrimAl 1.4.1 [85] run with ‘automated1’ and after further filtering with WitChi 0.1.0 [86] run with the quartic pruning algorithm in ‘touchdown’ mode.

Maximum-likelihood trees were inferred with IQ-TREE 2.4.0 [87] with the evolutionary model selected using ModelFinder [88]. Support values were determined using 1,000 replicates of both the SH approximate likelihood ratio test (SH-aLRT) [89] and ultrafast bootstrap (UFBoot) [90]. For the bac120 marker set, trees were also inferred using FastTree 2.1.11 [91] using (i) the full set of GTDB species representatives, MSA filtering as implemented in GTDB-Tk 2.4.1 and the WAG evolutionary model in order to replicate the tree inference method used by the GTDB [78] and (ii) the LG+G model with the MSA filtered using TrimAl and WitChi as described above in order to evaluate the impact of tree inference methods.

The tree shown in Fig. 1 was inferred over the full set of 215 GTDB species representatives and MGR MAGs as described above but pruned to contain only 1 genome per genus with a Latin name. The genome for the type species of the genus was retained when available.

### Inference of the *prfB* gene tree

*prfB* genes >180 bp were aligned with MAFFT 7.505 using the ‘auto’ argument. IQ-TREE 2.4.0 was used to infer the gene tree with ModelFinder used to select the most appropriate evolutionary model and support values determined using 1,000 replicates of SH-aLRT and UFBoot. The tanglegram between the *prfB* and species tree was generated with Dendroscope 3.8.10 [92].

### Identification of genes

MGR MAGs along with additional *Eggerthellaceae* genomes were annotated with Prokka 1.14.6 [93] using the bacterial reference databases. Aragorn 1.2.38 [94] was used internally by Prokka to identify tRNA genes. Prokka annotations were inspected to identify tRNA^Trp^ genes, peptide chain release factor 1 (RF1, *prfA*) and 2 (RF2, *prfB*) genes, genes comprising the *selABCD* operon and genes comprising the 16 S-23S-5S rRNA operon. The lack of pseudogenized *prfB* genes in the MGR-GC4 MAGs was established using a sequence similarity search. Specifically, a protein database of *prfB* genes from 61 *Eggerthellaceae* genomes in GTDB R10-RS226 (1 per genus) was constructed and TBLASTN 2.12.0+ [95] used to compare these protein sequences to the nucleotide sequences of the MGR MAGs. This resulted in full length hits with ~60% identity to the *prfB* genes in the 4 MGR-GC11 MAGs. By contrast, the 34 MGR-GC4 MAGs only had low sequence identity hits (~40%) that corresponded to the *prfA* gene in these genomes.

A differential gene presence analysis was performed by considering all genes identified by Prokka with a gene name and counting the number of genomes in each group with this gene (Table S5). Results are given for MGR-GC4 MAGs, MGR-GC11 MAGs and the four *Eggerthellaceae* genomes closest to the MGR clade (Fig. 1). Identification of motility and cell envelope genes were determined by inspecting Prokka annotations for specified gene names (Table S6).

### Alignment of conserved protein

Protein families where UGA aligned to a conserved tryptophan residue were identified using TIGRFAMs 17.0 [96] and HMMER 3.4 [97], with identified TIGRFAM families aligned with hmmalign. Conserved tryptophan residues were identified across the 38 MGR MAGs and genomes from *Eggerthella lenta* (GCF_000024265.1), *Adlercreutzia equolifaciens* (GCF_000478885.1) and *Phoenicibacter congonensis* (GCF_900169485.1). Alignments were screened to identify positions where a tryptophan residue was present in at least 90% of these 41 genomes and the 5 proteins most clearly illustrating the reassignment of UGA to tryptophan manually identified: TIGR00002 (*rpsP*; ribosomal protein bS16), TIGR00088 (*trmD*; tRNA (guanine(37)-N(1))-methyltransferase), TIGR00633 (*xth*; exodeoxyribonuclease III), TIGR01034 (*metK*; methionine adenosyltransferase) and TIGR01514 (*pncB*; nicotinate phosphoribosyltransferase).

### Calculation of genomic properties

Genomic properties were determined for 250 *Eggerthellaceae* genomes (Table S1) and genomes from taxa that use GC4 (Table 2). Specifically, genomes from GTDB R10-RS226, which is based on RefSeq release 226, were considered and subjected to the same quality-control (QC) criteria as used for the MGR MAGs. Notably, many species that use GC4 fail the GTDB QC criteria as they lack a sufficient number of phylogenetic marker genes due to their highly reduced genomes. Consequently, *Ca*. Hodgkinia cicadicola, *Ca*. Nasuia deltocephalinicola, *Ca*. Stammera capleta and *Ca*. Zinderia insecticola genomes were identified using NCBI classifications. Genomes belonging to the order *Mycoplasmatales* were identified using the GTDB taxonomy, and only genomes assembled from the type strain of a species were retained to focus on high-quality genomes. The %GC content and genome size of these species were determined using CheckM2, and the presence of tRNAs and the *prfB* gene identified using Prokka. The frequency of stop codon usage was determined by parsing annotated CDS features and tabulating the number of identified stop codons.

### Gene neighbourhood of *secA* gene

Prokka annotations were used to identify *secA* genes (see Identification of genes), and a custom Python script was used to create gene feature format (GFF) files with the 20 genes adjacent on either side of the *sec*A gene. These GFF files were provided to Clinker 0.0.31 [98] to generate the gene neighbourhood plot which was enhanced with visual elements using Inkscape.

### Host and geographic metadata

Host and geographic metadata was collated for 967 SRA samples where SingleM [38] predicted the abundance of CAVGFB01 or JAUNQF01 at >0.1% (Table S2). Sample metadata was obtained from the NCBI using the biosample2table.py script provided by the Jason Stajich Lab (https://github.com/stajichlab/biosample_metadata). The GTDB R10-RS226 SingleM database was supplemented with the 35 MGR MAGs, and the 967 samples were re-profiled to resolve the presence of taxa from the genera *Tapirivita, Equivita* and *Gorillivita* proposed in this study (Table S2).

## Supplementary material

10.1099/mgen.0.001767 Supplementary Material 1.

10.1099/mgen.0.001767 Supplementary Material 2.

## Abbreviations

*Ca.*: *Candidatus*
GC4: Genetic code 4
GC11: Genetic code 11
MAG: metagenome-assembled genome
MGR: mammalian gut reassignment
MSA: multiple sequence alignment
SH-aLRT: SH approximate likelihood ratio test
SRA: Sequence Read Archive
UFBoot: ultrafast bootstrap
